# Centers of excellence in healthcare institutions: what they are and how to assemble them

**DOI:** 10.1186/s12913-017-2340-y

**Published:** 2017-07-11

**Authors:** James K. Elrod, John L. Fortenberry

**Affiliations:** 1 Willis-Knighton Health System, 2600 Greenwood Road, Shreveport, LA 71103 USA; 2 LSU Shreveport, 1 University Place, Shreveport, LA 71115 USA

**Keywords:** Center of excellence, Integrated practice unit, Integrated healthcare delivery, Medical quality

## Abstract

**Background:**

Centers of excellence—specialized programs within healthcare institutions which supply exceptionally high concentrations of expertise and related resources centered on particular medical areas and delivered in a comprehensive, interdisciplinary fashion—afford many advantages for healthcare providers and the populations they serve. To achieve full value from centers of excellence, proper assembly is an absolute necessity, but guidance is somewhat limited. This effectively forces healthcare providers to pursue establishment largely via trial-and-error, diminishing opportunities for success.

**Discussion:**

Successful development of a center of excellence first requires the acquisition of a detailed understanding of the delivery model and its benefits. Then, concerted actions must be taken on a particular series of administrative and clinical fronts, treating them in prescribed manners to afford synergies which yield an exceptionally high level of care. To reduce hardships associated with acquiring this rather elusive knowledge, remedy shortcomings in the literature, and potentially bolster community health broadly, this article presents information and insights gleaned from Willis-Knighton Health System’s extensive experience assembling and operating centers of excellence. This work is intended to educate and enlighten, but most importantly, supply guidance which will permit healthcare establishments to replicate noted processes to realize their own centers of excellence.

**Conclusions:**

Centers of excellence have the ability to dramatically enhance the depth and breadth of healthcare services available in communities. Given the numerous mutual benefits afforded by this delivery model, it is hoped that the light shed by this article will help healthcare providers better understand centers of excellence and be more capable and confident in associated development initiatives, affording greater opportunities for themselves and their patient populations.

## Background

Healthcare providers face an interesting and extensive barrage of opportunities and challenges as they go about serving their patient populations. Among other things, financial resources often are in short supply, competition typically is robust, technologies are forever evolving, oversight bodies are demanding ever-increasing accountability, and patients are becoming more informed and desirous of the best care available [1–4].

In such complex and continually changing environments, some healthcare institutions are seeking to differentiate themselves by establishing niche programs focused on particular areas of medicine, delivering world class care and attention on these selected fronts [5]. Known as centers of excellence, these programs afford many advantages for healthcare institutions and their served markets [5, 6].

Perhaps most notably, centers of excellence distinguish institutions as citadels of expertise for the care and treatment of particular medical conditions, increasing opportunities to attract patients. They also have the ability to deliver enhanced quality through the application of innovative tools, technologies, and techniques which improve outcomes. Recruitment initiatives, too, are bolstered, permitting centers of excellence to assemble extensive reservoirs of skill and experience on clinical and administrative fronts. Further, centers of excellence can benefit institutions financially by increasing efficiencies and improving reimbursements [5–7].

To achieve full value from a center of excellence, proper assembly is an absolute necessity, but guidance is somewhat limited. Efforts published in the academic literature tend to focus narrowly on facets such as cost savings potential, impact on organizational dynamics, and the like (e.g., [8–11]). The trade literature supplies many accounts (e.g., [12–14]), but these often are too limited to supply substantive assembly guidance. Scarcity of assembly information is amplified further by the fact that centers of excellence represent key sources of competitive advantage. This naturally prompts at least some to refrain from circulating details as a means of preserving trade secrets [2, 4, 15, 16].

Without comprehensive assembly guidance, however, healthcare institutions desirous of establishing centers of excellence are effectively forced to do so largely via trial-and-error, diminishing their potential for success and reducing healthcare opportunities available in their communities. To reduce such hardships, remedy shortcomings in the literature, and potentially bolster community health broadly, this article presents information and insights gleaned from Willis-Knighton Health System’s extensive experience assembling and operating centers of excellence. This work is intended to educate and enlighten, but most importantly, supply guidance which will permit healthcare establishments to replicate noted processes to realize their own centers of excellence.

### Definition

Formally defined, a center of excellence is a program within a healthcare institution which is assembled to supply an exceptionally high concentration of expertise and related resources centered on a particular area of medicine, delivering associated care in a comprehensive, interdisciplinary fashion to afford the best patient outcomes possible. A type of integrated practice unit [1] and integrated healthcare delivery model [17], centers of excellence are essentially places where excellence on a particular medical front is delivered in a unique, focused manner to patients. Specialty areas frequently housed in centers of excellence include cardiology, orthopedics, oncology, ophthalmology, bariatric surgery, and neurology, just to name a few [5, 6, 18].

While one might assume that in the highly-regulated healthcare industry use of the center of excellence designation would be restricted to those providers which meet prescribed standards and hold associated certifications, this generally is not the case [19]. With few exceptions, the center of excellence designation can be applied at will by healthcare establishments [18, 19]. This freedom, however, must be used responsibly, with healthcare institutions taking great care to apply the designation only in cases where warranted. It must be much more than a marketing slogan [20, 21].

In terms of presentation, the application of the center of excellence designation varies. Some institutions actively promote it by including the designation in the formal brand name of the entity (e.g., Knightsbridge Center of Excellence for Cardiovascular Health), while others do so more subtly, listing it as a secondary reference underscoring the brand name of the particular establishment (e.g., Meadowbrook Heart Clinic, A Center of Excellence in Cardiovascular Care). Some even forego active promotion altogether and instead simply operate the associated model and let the quality delivered ultimately serve as the promotional mechanism. Notably, even the naming of the institutional structures that house centers of excellence varies from facility to facility and sometimes even within given healthcare establishments. Some are labeled centers, others are referred to as institutes, still others identify themselves simply as departments, and so on. While these various structures technically are distinctive, in practice, many institutions use the terms interchangeably, with selections being based on institutional preferences [18, 22].

Given the differences in application of the center of excellence designation and varying institutional structures which house these centers, it is apparent that, at least in thoughtfully assembled and well-operated examples, their root value rests less in their promotional potential and more in their ability to deliver enhanced healthcare experiences. This is in keeping with commonly observed missions of healthcare institutions which routinely emphasize patient care above all other elements of operation [23–25].

To further bolster an understanding of centers of excellence, it is helpful to examine real-world operations for associated enlightenment. One particular healthcare institution with extensive experience developing and operating centers of excellence is Willis-Knighton Health System.

### Willis-Knighton Health System and centers of excellence

Willis-Knighton Health System is a nongovernmental, not-for-profit healthcare provider based in Shreveport, Louisiana. Delivering comprehensive health and wellness services through multiple hospitals, numerous general and specialty medical clinics, an all-inclusive retirement community, and more, the system holds market leadership in its served region, centered in an area known as the Ark-La-Tex, where the states of Arkansas, Louisiana, and Texas converge. Decades ago, however, the establishment occupied a much less prominent position and executives began searching for avenues to expand and improve its footprint. With a keen understanding that quality above all else attracts patients, innovative delivery models were explored in an effort to identify opportunities which would afford quality enhancements that in turn would attract increasing numbers of patients. Centers of excellence emerged as an option and, on further investigation, executives found the model to make sense on myriad levels, prompting associated pursuits. Willis-Knighton Health System’s first centers of excellence, originating in the 1980s, proved the viability of the delivery model, leading to the development of others. It now operates 11 centers of excellence, as follows.

- WK Cancer Center
- WK Center for Orthopedics
- WK Center for Reproductive Medicine
- WK Center for Women’s and Children’s Health
- WK John C. McDonald Regional Transplant Center
- WK Laparoscopic and Robotic Surgery Center
- WK Metabolic Surgery Center
- WK Stroke Center
- WK Eye Institute
- WK Heart and Vascular Institute
- WK Rehabilitation Institute

The system uses “center” for operations working in especially concentrated areas and “institute” for more broad-based offerings, with these typically containing expanded service arrays within disciplinary lines and involving significant research components. Both are structured in accordance with the center of excellence delivery model. The center of excellence designation is not used in marketing; Willis-Knighton Health System operates the model for the level of care that it produces which in turn serves as the primary promotional mechanism. The system considers the center of excellence delivery model to be a vital component of its service mix and credits it with facilitating growth that led to its current market leadership position.

### Distinguishing features

Over its many decades of experience operating multiple centers of excellence, combined with examinations of peer centers of excellence and ongoing reviews of relevant findings in the literature, Willis-Knighton Health System has identified six particular fronts—organization design, servicescape design, personnel, medical care, marketing, and finance—that distinguish centers of excellence from traditional healthcare delivery models. The system has observed that when each of these fronts is treated in prescribed manners, synergies between and among the components emerge which yield an exceptionally high level of care largely exceeding that delivered in traditional settings. These fronts, illustrated using examples from Willis-Knighton Health System’s centers of excellence, are described as follows.

#### Organization design

Organization design refers to the manner in which work responsibilities and resources are divided and allocated to units in an institution to ensure coordination and performance, permitting mission fulfillment [26]. In a center of excellence, work responsibilities and resources associated with addressing a particular medical condition are centralized into a functional organizational subunit with responsibilities for delivering the full continuum of care, often within a single medical building [1, 5–7]. Selection of the particular organizational structure (e.g., matrix, program/service line, etc.) is dependent on the organizing preferences of given institutions.

**Fig. 1:**
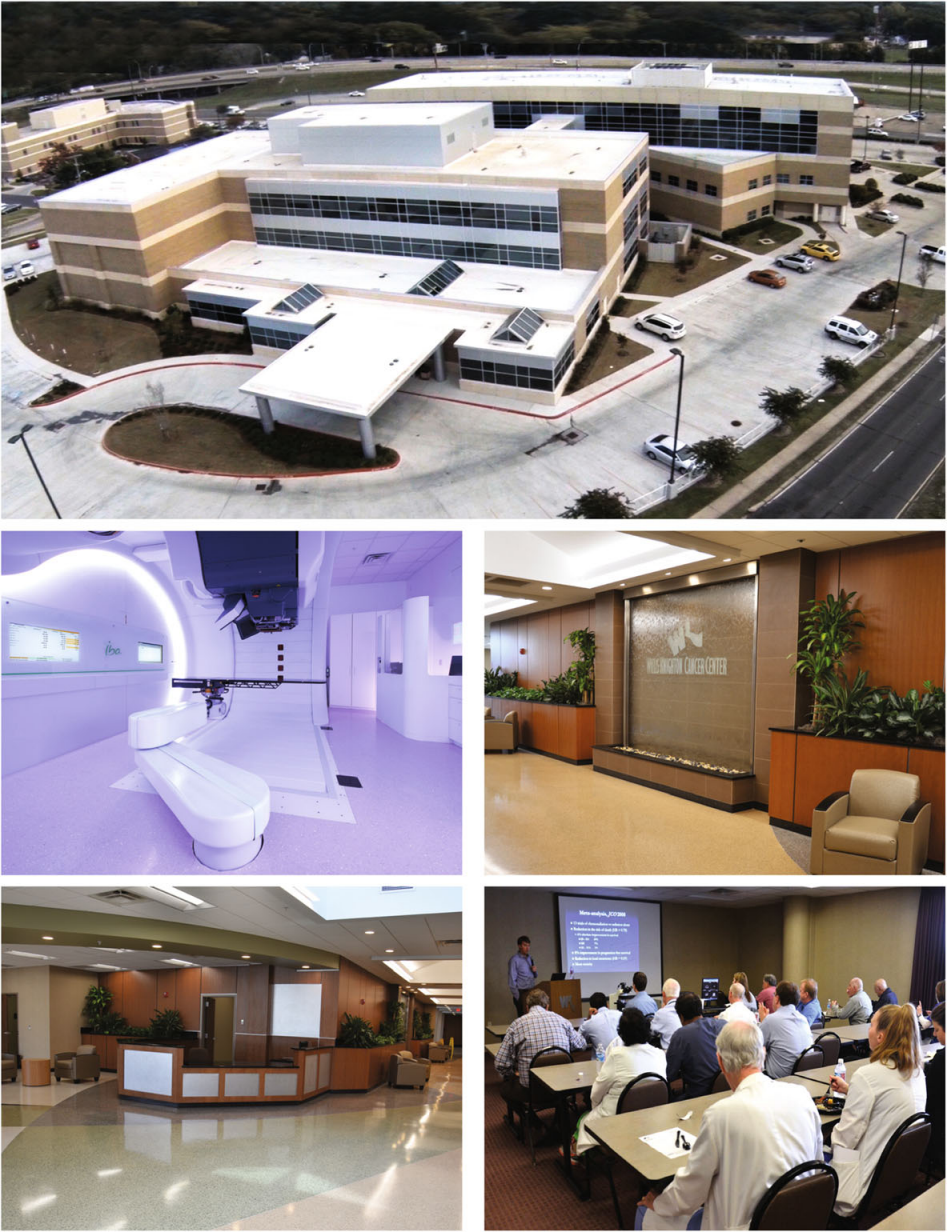
The WK Cancer Center: sights and scenes. Copyright © 2016 Willis-Knighton Health System. Used with permission. The WK Cancer Center is one of 11 centers of excellence operated by Willis-Knighton Health System. At a single site on the system’s main campus, cancer patients can receive virtually every service associated with their care. The customized servicescape features an attractive, user-friendly layout and serene atmosphere which includes comfort-minded appointments, like a peaceful water wall. Expert medical professionals conference regularly to advance care which they deliver using the latest technologies, including the ProteusONE which delivers proton therapy via a pencil beam scanning unit, offering the most precise form of radiation therapy available. The WK Cancer Center exemplifies the application of the center of excellence delivery model

As an example, the WK Cancer Center, profiled in Fig. 1, offers patients the convenience of visiting a single site on Willis-Knighton Health System’s main campus for receipt of every service related to their care and treatment, from patient education seminars to social services to pharmacy to chemotherapy to one of the most advanced cancer treatment technologies available, proton therapy. This stands in sharp contrast to traditional delivery approaches where care components from myriad departments are strung together to effect medical interventions, often requiring patients to visit multiple buildings on campuses or even multiple locations within communities to realize the continuum of care. Centers of excellence also feature shared governance systems, characterized by transparency, that foster collaboration across disciplinary lines and ensure joint accountability for outcomes [5, 6, 27, 28].

The very tight, centralized organization designs used in properly assembled centers of excellence foster many of the benefits associated with this particular delivery model. Patient convenience is maximized by consolidating previously dispersed care components, improving satisfaction. The formally-structured, concentrated focus on particular medical conditions permits specialization, affording tailored care experiences which feature customized environments and expert personnel. The centralized structure also pools resources which otherwise would be distributed more broadly, with this fostering efficiencies and effectiveness [29, 30].

As a multihospital system, Willis-Knighton Health System could choose to offer cardiovascular surgery services, for example, at multiple locations across its served region, but this broadly distributed approach is highly inefficient and also negatively impacts patient outcomes. By consolidating specialty offerings into strategically-located centers of excellence—in this particular case, the WK Heart and Vascular Institute—Willis-Knighton Health System achieves vital economies of scale which reduce costs and permit more resources to be directed toward patient care. Further, rather than fielding multiple dispersed patient care teams which perhaps would conduct dozens of cardiovascular operations annually, its single, central team has opportunities to conduct hundreds of operations (597 cardiovascular surgeries were performed in 2016), increasing quality and improving outcomes.

#### Servicescape design

The term servicescape refers to the collection of elements which compose the service environment of an institution. Common servicescape components include architecture, parking, signage, equipment, technology, ergonomics, ambiance, and much more [15]. Upgraded materials, design, and workmanship characterize the servicescapes of properly assembled centers of excellence, carrying the excellence theme across all features of the environment. While these sorts of upgrades are common in most any quality healthcare establishment, centers of excellence take a critical additional step, customizing entire servicescapes to serve patients experiencing the particular medical conditions addressed by the given centers [14, 31, 32].

Willis-Knighton Health System’s Metabolic Surgery Center, for example, incorporates furnishings, fixtures, and equipment specifically designed to accommodate obese patients, even placing attention on elements used by the family members of these patients, as they very often are themselves overweight. The tools and technologies used to treat obese patients are state-of-the-art, market leading offerings. Further, staff members receive sensitivity training to ensure that they understand and can relate to people struggling with weight control issues. Such investments ultimately afford patient experiences customized to address the specific needs of populations suffering from targeted maladies. Since centers of excellence concentrate on particular medical conditions, associated servicescapes can focus fully on the selected fronts, yielding tailored environments that are not possible in settings which address a wide range of medical conditions.

#### Personnel

One of the most notable features of centers of excellence pertains to the depth and breadth of qualifications possessed by their personnel. Indeed, extensive reservoirs of skill and experience reside within centers of excellence and, in keeping with the specialization characterizing this delivery model, the skills possessed by staff members specifically pertain to the medical conditions addressed by the given centers. These experts are assembled via carefully-planned organizational structures into collaborative, interdisciplinary teams and directed in a manner to deliver exceptional care, something facilitated by open communication, including formal opportunities to share experiences [5, 6, 27, 28]. Centers of excellence typify what organizational behaviorists refer to as learning organizations whereby institutional members actively share knowledge, insights, and experiences to improve understanding and initiate enhancements resulting from lessons learned [33].

The WK Cancer Center, for example, holds regular cancer conferences which facilitate active integration of expertise, mutual learning, concerted problem solving, and continuous improvement. Opportunities for engagement go beyond that which is required by the center’s accrediting bodies to address areas of special interest to staff members. These sorts of collaborative learning experiences are commonplace in all of Willis-Knighton Health System’s centers of excellence and they set the stage for best practices to not only be embraced but also advanced.

Notably, the unique properties afforded by centers of excellence make them highly attractive to job seekers, bolstering the ability of institutions to recruit the best physicians, nurses, administrators, and technicians in the marketplace, affording a significant competitive advantage. The latest technologies, a mainstay in well-designed centers of excellence, are particularly attractive to physicians seeking to exercise their skill sets assisted by the most advanced equipment available [5–7].

As one of Willis-Knighton Health System’s first centers of excellence, the WK Eye Institute, which was established in the 1980s, invested in cutting edge technologies on its introduction. This set the stage for the system to recruit and retain practitioners with market leading skill sets, a pattern affording numerous advancements and innovations. For example, the WK Eye Institute was the first establishment in the market to offer foldable silicone intraocular lens implantation services, no-stitch cataract surgery using only local anesthesia, LASIK surgery, and YAG laser procedures. It continues to deliver the market’s leading ophthalmology services, namely including laser-assisted cataract surgery using the LenSx laser and refractive surgery using the WaveLight system, with these tools permitting Willis-Knighton Health System to attract and retain the best and brightest who apply their advanced skill sets to benefit patients.

#### Medical care

Well-equipped servicescapes and expert workforces converge via effective organization designs to permit a level of medical care that’s difficult to match outside of the center of excellence delivery model. These various elements are carefully woven together to form synergies which yield an integrated, comprehensive continuum of care designed to support patients from their initial presentation through to completion of service delivery. Medical care isn’t delivered in assembly line fashion but instead is customized to address the specific wants and needs of individuals experiencing targeted health matters [1, 5, 6].

Possessing a deep understanding of its patient populations, the WK Cancer Center strives to expedite consultations with oncologists, with a standing rule being that appointments are scheduled within 48 h of initial contact. Staff members realize the agonizing stress associated with a cancer diagnosis, something hastened by delays in getting answers. Expedited consults help to reduce at least some of the burdens experienced by patients during such difficult times, with this illustrating one of many facets of the WK Cancer Center’s tailored approach to care.

Interestingly, Willis-Knighton Health System has observed that its centers of excellence positively impact care provided even outside of its designated centers. Although efforts are made to centralize and compartmentalize the services of the system’s centers of excellence at single sites, there invariably are times when center patients must engage external units. Patients of the WK Stroke Center, for example, might experience health events that necessitate emergency care, taking them to one of Willis-Knighton Health System’s emergency departments. Because of this, center of excellence leadership teams actively engage the leaders of various external units to ensure that excellence is delivered without fail across the entire patient experience. This has the effect of elevating service quality system wide, affording a stronger core institution.

#### Marketing

Properly assembled and operated centers of excellence establish healthcare providers as marketplace authorities on particular medical fronts, supplying helpful marketing benefits. With proper marketing efforts directed toward promoting the depth and breadth of services provided, combined with excellent care delivery which generates positive word-of-mouth communications from patients, centers of excellence effectively create ongoing top-of-mind awareness which has the effect of bolstering patient volume [34].

Centers of excellence are particularly effective for purposes of product differentiation, the practice of embedding distinguishable features and benefits into given products (i.e., goods and services) in an effort to differentiate them from competitive offerings and foster customer recognition [15]. The more competitive the industry, the more essential product differentiation becomes [4, 15, 16]. Given the intense rivalry that characterizes the healthcare industry, efforts naturally must be taken to effectively distinguish service offerings from those of marketplace rivals and centers of excellence provide significant opportunities to do just that.

Importantly, Willis-Knighton Health System has observed that its centers of excellence, courtesy of their high-profile status, provide a halo effect which positively impacts all of its service lines [34]. Their high-attraction capabilities also afford opportunities to cross-sell services outside of given centers of excellence, as patients very often have more than one medical need. These features position centers of excellence as market share boosters, a point that certainly shouldn’t be overlooked by healthcare executives, given the competitive state of the industry. As noted earlier, Willis-Knighton Health System attributes its achievement of market leadership largely to the center of excellence delivery model which dramatically hastened market share growth.

#### Finance

Centers of excellence have great potential to improve the financial performance of healthcare entities [1, 5, 6]. This benefit ultimately is derived from the collection of other benefits afforded by these centers. Product differentiation hastens patient volume which positively impacts bottom line performance. Quality enhancements attract patients seeking the best care possible, bolster patient satisfaction and positive word-of-mouth communications, reduce the potential for malpractice lawsuits, and facilitate the attainment of standards required to maximize reimbursements under models such as value-based purchasing and bundled payments. Additionally, the concerted direction of resources toward highly-specialized areas of care, often centralized at single sites, permits the achievement of economies of scale, further generating savings and improving financial performance.

Willis-Knighton Health System has witnessed firsthand the synergies afforded by centers of excellence and their positive impact on financial performance. Despite the fantastic expenses associated with establishing centers of excellence, the mutual benefits afforded, including financial ones, provide ample justifications for their pursuit.

### Establishing centers of excellence

Over its many experiences establishing and operating centers of excellence, Willis-Knighton Health System has acquired significant associated proficiencies. Those pertaining to the assembly of centers of excellence are perhaps most critical as they set the stage for operations going forward. Efforts must be on the mark throughout the development process as errors experienced here will plague operations potentially indefinitely and could even result in failures. To ensure consistency in assembly and increase prospects for success, Willis-Knighton Health System crafted a framework, known as the Center of Excellence Establishment Protocol, to guide center of excellence development initiatives. Completion of the 3-stage protocol yields an operational center of excellence. Illustrated in Table 1, the framework is explained as follows.

**Table 1:** The center of excellence establishment protocol

Stage 1: vision and validation
*When considering the establishment of a center of excellence, conduct a series of initial assessments to conceptualize the offering and ascertain feasibility*
a. Appoint an interdisciplinary committee charged with envisioning the prospective center of excellence
b. Assess the availability of foundational requirements for success by verifying the sufficiency of financial resources, organizational culture, and leadership support
c. Craft working mission and vision statements for the prospective center of excellence
d. Conduct a feasibility study to assess community need, determine services to be featured, estimate patient volume, and ascertain the financial viability of the proposed center
Stage 2: design and development
*With conceptualization completed and feasibility verified, prepare detailed plans which address each component of the center of excellence*
a. Organization design
i. Prepare a comprehensive organizational chart which depicts positions and associated reporting relationships required for comprehensive, single-site treatment of targeted medical conditions
ii. Devise shared governance mechanisms and processes to ensure transparency and accountability
b. Servicescape design
i. Aided by field trips to peer centers, insights from internal and external experts, and accounts in publications, design a service environment customized to address the needs of patients facing the medical conditions targeted by the proposed center
ii. Determine the assets to be housed within the given center, the anticipated patient volume, the accommodations required by staff members, and the associated spatial requirements necessary to deliver the entire continuum of care within the servicescape
iii. Identify an appropriate site to house the center of excellence and work with architects, engineers, designers, and other professionals to prepare formal plans
c. Personnel
i. Determine staffing requirements and the specific qualifications (e.g., credentials, skills, experience) needed to fulfill the center’s mission
ii. Formulate an associated recruitment plan to acquire highly qualified personnel
d. Medical care
i. Formulate plans to ensure that servicescape and workforce assets are carefully integrated via the organization design to yield outstanding medical care and attention
ii. Incorporate organizational learning principles to facilitate best practices, continuous improvement, and innovation
iii. Envision which areas outside of the center’s command and control patients likely will encounter so that relationships can be formed to facilitate the delivery of excellence across the entire patient experience
e. Marketing
i. Select the center’s brand name, design brand elements (e.g., logos, slogans), and formulate an associated marketing communications plan and, ideally, a center-specific marketing plan
ii. Envision potential opportunities to cross-sell services to patients
f. Finance
i. Investigate opportunities to maximize efficiencies and bolster reimbursements and work to incorporate these into clinical and administrative processes to enhance revenue
ii. Ensure that synergies between and among the distinguishing features of the center are maximized to afford enhanced financial performance
Stage 3: completion and commercialization
*On approval of design and development plans, the center of excellence moves from the blueprint stage to construction and then launch, concluding the establishment protocol*

#### Stage 1: vision and validation

When considering the establishment of a center of excellence, intensive efforts first are directed toward conceptualizing the offering and ascertaining its feasibility. This begins by appointing an interdisciplinary committee charged with visualizing and vetting the proposed center of excellence. Committee composition is dependent on the focus of the given center of excellence, but generally includes representation from the occupational categories required to operate the center (e.g., administrators, physicians, nurses, technicians). External parties, such as consultants, architecture and engineering experts, design professionals, peer institution leaders, and community stakeholders, are scheduled to attend committee meetings as needed for provision of associated insights and expertise.

Before focusing on the specifics associated with the center, committee members must first assess the institution’s readiness to operate the given center by verifying the sufficiency of financial resources, organizational culture, and leadership support. Even with only a cursory review of the attributes characterizing centers of excellence, it goes without saying that extensive financial resources are required to establish these programs. Such expenditures, however, are forwarded in anticipation of the greater gains afforded when operations ensue. Regardless, access to sufficient capital is required in order to successfully establish centers of excellence, something the assigned committee will need to investigate and verify before pursuits begin in earnest.

The organizational culture of a healthcare establishment exerts a profound influence on virtually every facet of its operation. It can be positive, fostering helpful attitudes, teamwork, and productivity, but it also can be negative, harming institutional operations [35, 36]. Culture flaws or the presence of negative subcultures scattered about the institution can have a detrimental impact on centers of excellence. Even as somewhat self-contained entities within greater organizations, symbiotic relationships invariably exist. As such, committee members must ensure that the culture of the given institution is one which will support the sound establishment and productive operation of the proposed center.

The costs and complexities associated with centers of excellence require extensive initial and ongoing support on the part of the institution’s leadership. Commitment from top leaders obviously is required for program authorization, but this dedication must be sustained in order for centers of excellence to endure. Additionally, commitment from departmental leaders across the institution is necessary, as external units at least occasionally will be called upon to support aspects of the continuum of care delivered by the center. Ultimately, comprehensive support across all units that will directly or indirectly support the patient care initiatives provided by the center must be acquired for best results.

Assuming the presence of sufficient financial resources, a positive organizational culture, and comprehensive leadership support, efforts of the committee are then directed toward crafting working mission and vision statements. This helps to conceptualize the role that the center of excellence will play in the marketplace, the populations that it will address, and the benefits that it is expected to afford. Mission and vision statement assembly activities also present opportunities to ensure that prospective centers are sufficiently differentiated from other service offerings in given marketplaces.

On development of these items, a general conceptual framework emerges which in turn must be validated via a feasibility study designed to assess community need, determine the specific array of healthcare services to be featured, estimate patient volume, and ascertain through associated financial analyses, including revenue projections, the viability of the proposed center. If results do not support the center’s establishment, the pursuit should be abandoned or perhaps reformulated to remedy whatever shortcomings were discovered, potentially bringing the idea into the realm of feasibility. If results support the establishment of the center of excellence, pursuits can advance to the design and development stage.

#### Stage 2: design and development

With conceptualization completed and feasibility verified, detailed plans are prepared which address the components of organization design, servicescape design, personnel, medical care, marketing, and finance, treating these in defined manners called for by the center of excellence delivery model.

Addressing the organization design component begins with the assembly of a comprehensive organizational chart which identifies positions and associated reporting relationships necessary to support the center of excellence. Care must be taken to design the structure in a manner compliant with the tenets of the delivery model, ensuring a centralized, integrated arrangement which addresses the entire continuum of care required by patients. Shared governance mechanisms, components designed to foster interdisciplinary teamwork by giving a voice to those involved in delivering the services provided by the center, must also be determined and structured. Oversight committees, for example, must be assembled to ensure representation from clinical, administrative, and patient ranks. Leadership structures must also be determined, with collaboration, especially between administrative and clinical areas, being essential. Physicians, in particular, must be engaged to ensure high involvement in operational processes and procedures to advance excellence in service delivery. Operations must be conducted in a transparent manner characterized by open communication between and among parties. Accountability for assigned processes and outcomes must be required of all roles.

Servicescape formulation best begins by touring operating centers of excellence which are addressing the proposed center’s targeted medical conditions. Such tours can generate a wealth of ideas and they also can help institutions establish peer relationships which can yield mutual benefits. Beyond such tours, in-house experts, accounts from scholarly and trade publications, and architecture, engineering, technology, and design professionals must be consulted for ideas, insights, and guidance in the assembly of the center’s patient-centered service environment.

Ultimately, determinations must be made regarding the assets to be housed within the given center, the anticipated patient volume, the accommodations required by staff members, and the associated spatial requirements necessary to deliver the entire continuum of care within the servicescape. With those requirements stipulated, site selection and acquisition activities can be conducted, after which architects, engineers, designers, and related professionals can then prepare formal plans.

The personnel requirements of the center of excellence must be determined and specific qualifications (e.g., credentials, skills, experience) must be stipulated. It is especially important to staff the center with personnel who possess market leading qualifications, with a premium being placed on acquiring candidates with specialty credentials demonstrating prowess in addressing the specific medical conditions targeted by the given center. Importantly, special attention must be directed toward positions envisioned to be difficult to fill, with proper resources being allocated to ensure recruitment success. With knowledge of staffing requirements in hand, internal and external recruitment plans are developed to ensure that properly qualified personnel in sufficient numbers are available for service when the center of excellence opens.

With medical care of exceptional quality being the ultimate distinctive point associated with centers of excellence, plans must be formulated to ensure that servicescape and workforce assets are carefully integrated via the organization design to yield outstanding medical care and attention. Centers must incorporate best practices at every possible opportunity, initiate mechanisms to ensure continuous improvement, and pursue innovations which benefit patients and institutions alike. Since centers of excellence exist within greater healthcare institutions, efforts must also be directed toward envisioning which areas outside of the center’s command and control patients likely will encounter so that relationships can be formed to facilitate the delivery of excellence across the entire patient experience.

Marketing aspects to be addressed during the design and development stage primarily pertain to branding and related marketing communication matters. First and foremost, the brand name of the center of excellence must be determined. Will the center of excellence descriptor be used and, if so, will it be part of the entity’s formal brand name or simply serve as a secondary reference underscoring the name of the establishment? With that particular determination made and other brand design activities (e.g., logo and slogan development) completed, broader marketing communication matters can be addressed, including introductory advertising, grand opening ceremony plans, and so on. Being a component within a larger healthcare institution, a newly established center of excellence normally will fall under the existing marketing plan operated by the given institution. Regardless, a center-specific marketing plan is advised, especially during the early stages of the center’s life. Since the center likely will attract a high degree of attention and accompanying patient volume, efforts also should be directed toward ascertaining logical tie-ins with other service lines in order to identify opportunities to cross-sell services to patients.

Regarding matters of finance, many of the features of centers of excellence naturally afford opportunities which positively impact financial performance, but concerted attention generally is required in order to realize them. Standardization of processes, for example, can generate efficiencies which reduce waste and improve performance. Centralizing operations also affords opportunities to generate efficiencies. Further, resulting quality metrics can boost reimbursement potential, offering another opportunity to bolster the financial position of the center of excellence. Cost savings, efficiencies, economies of scale, and other value-laden avenues all must be consciously and tactfully engineered in order to garner maximum returns. As such, all development activities must be viewed with an eye toward financial impact as a means of ensuring that efficient and effective processes that support financial viability are incorporated at each and every turn.

#### Stage 3: completion and commercialization

With the design and development stage completed, plans are submitted for approval. On authorization, the center of excellence moves from the blueprint stage to construction and then launch, concluding the establishment protocol. The unit then falls under the auspices of the prevailing strategic management framework of the greater healthcare institution which includes standard processes for planning, implementation, and evaluation activities to help ensure that the newly formed center of excellence operates proficiently and achieves designated goals.

### Operational considerations

Throughout the Center of Excellence Establishment Protocol, as with most comprehensive undertakings, a premium is placed on making wise decisions based on accurate information. Proper expertise is vital for achieving the best outcomes. In Willis-Knighton Health System’s case, executives worked over many years to develop internal expertise and nurture relationships with external parties (e.g., consultants, architecture and engineering firms, peer healthcare institutions) for provision of supplementary insights. These efforts ultimately yielded a significant competitive advantage which fostered success across all of its center of excellence development initiatives, with returns on this investment continuing to this day.

Healthcare institutions desiring outcomes similar to those achieved by Willis-Knighton Health System should follow suit by developing knowledge resources of their own to guide associated endeavors. While acquisition of such can take time, especially for newly established healthcare facilities, this shouldn’t discourage pursuit of centers of excellence. As internal expertise is being accumulated, institutions can look to external parties to supply required insights, providing a helpful bridge for addressing any knowledge gaps encountered, thus permitting complex initiatives to move forward.

## Conclusions

Willis-Knighton Health System’s experiences involving the establishment and operation of centers of excellence have been highly rewarding on multiple levels. Perhaps most importantly, these initiatives and efforts have enhanced the depth and breadth of healthcare services available in the communities served by the system. Without question, centers of excellence require significant resources, but if well designed and developed, such expenditures represent investments that have the potential to pay dividends that could even be considered to be priceless, especially in cases where in the absence of given centers, communities would simply have to do without needed healthcare services.

The Center of Excellence Establishment Protocol presented in this article supplies helpful guidance that can be used by any healthcare institution desirous of pursuing centers of excellence. Since resources which offer detailed assembly information appear to be limited, it is hoped that the light shed by this article will help healthcare providers better understand centers of excellence and be more capable and confident in associated development initiatives, affording greater opportunities for themselves and their patient populations.
